# Three-dimensional structured-light robot-assisted frameless versus frame-based stereotactic brain biopsy: a retrospective study of 54 patients

**DOI:** 10.3389/fneur.2026.1758309

**Published:** 2026-04-15

**Authors:** Liangchen Zhong, Zhenyu Luo, Dengfeng Wan, Zhaomu Zeng, Jianzhong Zhang

**Affiliations:** Department of Neurosurgery, Jiangxi Provincial People’s Hospital, The Affiliated Hospital of Nanchang Medical College, Nanchang, China

**Keywords:** 3-dimensional structured light, brain biopsy, glioma, stereotactic brain biopsy, surgical robot

## Abstract

**Introduction:**

This study was performed to evaluate the surgical efficiency, localization accuracy, biopsy success rate, and operative safety of three-dimensional (3D) structured-light robot-assisted stereotactic brain biopsy using the Huake Precision SR1-3D system.

**Methods:**

Clinical data were retrospectively collected from 54 patients who underwent stereotactic brain biopsy in the Department of Neurosurgery, at Jiangxi Provincial People’s Hospital between February 2017 and November 2022. Among them, 27 patients underwent frame-based stereotactic brain biopsy using the Anke system, and 27 patients underwent robotic-assisted frame-less stereotactic brain biopsy using the Huake Precision SR1-3D structured-light robot. The registration error of the Sinovation software based 3D structured-light facial scan was assessed. Operative time, diagnostic yield, incidence of adverse surgical events, length of hospital stay, surgical costs, entry-point error, and target-point error were compared between the two groups.

**Results:**

A total of 54 patients were analyzed, including 27 in the robot-assisted group and 27 in the frame-based group. The mean registration error of the 3D structured-light facial scan was 0.31 ± 0.07 mm. The mean operative time in the robot-assisted group was significantly shorter than that in the frame-based group (*p* < 0.05). No significant differences were observed in diagnostic yield, postoperative adverse event rate, or length of hospital stay between the groups (all *p* > 0.05). The surgical costs were higher in the robot-assisted group than in the frame-based group (*p* < 0.05). The robot-assisted group exhibited smaller entry-point and target-point errors compared to the frame-based group (both *p* < 0.05).

**Conclusion:**

This study found that, 3D structured-light robot-assisted stereotactic brain biopsy is associated with shorter operative times and reduced localization error compared to frame-based stereotactic brain biopsy, demonstrating favorable precision and procedural efficiency.

## 1 Introduction

Stereotactic brain biopsy was first implemented in 1908 and has a history of more than a century (1). Compared with conventional biopsy techniques, stereotactic methods provide greater precision and safety (2). However frame-based stereotactic approaches have notable drawbacks, including prolonged preoperative preparation and the requirement for imaging with a rigid metal frame in place, which may impose a psychological burden on patients (3). Additionally, human errors in calculating three-dimensional (3D) target coordinates and in intraoperative coordinate verification can impact surgical accuracy and outcomes. In 1985, YikSan Kwoh performed the first robot-assisted stereotactic brain biopsy using the PUMA 200 system, marking the initial application of robotic technology in neurosurgical biopsy (4). Since then, research on surgical robots has expanded rapidly, accompanied by increasing clinical applications (5). Robot-assisted stereotactic techniques eliminate the need for computed tomography (CT) with a metal head frame by enabling preoperative image fusion for trajectory planning and using to plan puncture trajectories and employing robotic arms to assist execution, thereby shortening operative time, improving patient experience, and enhancing procedural flexibility (6). To date, multiple countries have developed robotic systems for stereotactic brain biopsy, including the PUMA 200, Neuromate, and Pathfinder platforms (7). Among these systems, the ROSA surgical robot has emerged as a representative device for stereotactic brain biopsy due to its minimally invasive, safe, efficient, and accurate characteristics (8).

3D structured light technology is most commonly applied in facial recognition systems in smart devices, such as smartphones. Its primary function is to calculate the spatial positions and depth of the objects, thereby reconstructing 3D space (9). The Huake Precision SR1-3D surgical robot is the world’s first surgical robotic system to integrate 3D structured light technology with HoloShot intelligent sensing. Within tens of seconds, the system can capture depth and positional information for millions of surface and environmental features of an approximately complete cranium, substantially improving both the efficiency and accuracy of patient registration. In addition, its enhanced environmental perception capability eliminates the discomfort and inconvenience associated with rigid head-frame fixation. The system supports advanced intelligent functions, including automatic image registration, automatic patient registration, and automatic robotic-arm positioning (10).

This study retrospectively reviewed the clinical data of patients who underwent stereotactic brain biopsy in the Department of Neurosurgery at Jiangxi Provincial People’s Hospital between February 2017 and November 2022, in order to evaluate the operative efficiency, targeting accuracy, biopsy success rate, and safety of stereotactic brain biopsy assisted by the Huake Precision SR1-3D robotic system.

## 2 Materials and methods

### 2.1 General information

A retrospective analysis was conducted on 54 patients who underwent stereotactic brain biopsy using domestically produced Anke frame system or the Huake Precision SR1-3D surgical robot at the Department of Neurosurgery, Jiangxi Provincial People’s Hospital, between February 2017 and November 2022. For patients who underwent brain biopsy before November 2020, robotic equipment was unavailable; therefore, all procedures were performed using the conventional frame - assisted technique. These patients were assigned to the frame-based group, with a total of 24 cases. A total of 30 patients were treated after the equipment was officially put into clinical application (from November 2020 to November 2022). They were preferentially assigned to the robot-assisted group (*n* = 27). The frame-based technique was used as an alternative only when the robotic system malfunctioned or when a patient declined robotic assistance; three such cases occurred. This study was approved by the Ethics Committee of Jiangxi Provincial People’s Hospital.

Inclusion criteria were as follows: (1) imaging evidence of an intracranial lesion for which a definitive diagnosis could not be established; (2) imaging findings indicating lesions located in deep functional brain regions or multifocal/diffuse lesions unsuitable for direct surgical resection; (3) patients who were generally unfit for craniotomy and require histopathological diagnosis to guide subsequent treatment decisions.

Exclusion criteria included: (1) inability to tolerate stereotactic biopsy; (2) severe coagulation disorders or hemodynamic instability; (3) incomplete clinical data.

### 2.2 Methods

#### 2.2.1 Preoperative imaging

All patients underwent preoperative cranial Magnetic Resonance Imaging (MRI) (3.0 T, thin-slice scanning with a slice thickness of 1.0 mm) or cranial CT examination (conventional CT, 1.0 mm slice thickness). A postoperative cranial CT scan (scanning range from below the nasal tip to the cranial vertex skin) was performed on postoperatively day 1.

For patients in the Huake Precision SR1-3D surgical robot group, MRI data were acquired 1 week prior to the procedure. To optimize image quality, all MRI examinations were performed using a 3.0 T scanner with 1.0 mm thin-slice acquisition, including 3D T1-weighted and T2 -weighted fluid-attenuated inversion recovery (FLAIR) unenhanced sequences, contrast-enhanced T1-weighted imaging, and additional specialized sequences such as diffusion tensor imaging and magnetic resonance venography Preoperative MRI data were obtained in Digital Imaging and Communications in Medicine (DICOM) format.

A domestically manufactured Anke head frame was applied under local anesthesia for stereotactic frame-based procedures, perform followed by cranial CT scanning. The lesion was positioned as close as possible to the center of the frame. CT images were acquired using continuous helical scanning with a slice thickness of 1.0 mm, with the baseline plane parallel to the frame. Obtain The CT data were obtained in DICOM format.

All patients underwent a CT scan on postoperative day 1 (conventional CT, slice thickness 1.0 mm, scan range from below the nasal tip to the scalp at the cranial vertex).

#### 2.2.2 Preoperative planning

Huake Precision SR1-3D surgical robot group: After entering the surgical planning interface, all image sequences were registered to the reference sequence. Planned trajectories were designed to avoid areas with dense vasculature; when complete avoidance was not feasible, a minimum spatial distance of greater than 3 mm between the trajectory and the nearest vessel was ensured. The entry trajectory was designed to be as perpendicular to the skull surface as possible to minimize drill slippage, as a reduced entry angle also improves puncture accuracy. For trajectories with a short intracranial distance, the skin entry point was ensured to be at least 10 mm away from the scalp surface. Potential collisions along the planned 3D trajectory were carefully evaluated and avoided.

Frame-based group: Under local anesthesia, a domestically produced Anke stereotactic head frame was applied, and the lesion was positioned as close as possible to the center of the frame. Cranial CT scanning was performed using continuous helical scanning with 1 mm slice thickness, with the baseline plane parallel to the frame. The CT DICOM data were imported into the Anke stereotactic biopsy planning system. An appropriate puncture trajectory was designed, and the entry point and target point were determined. The stereotactic system was then used to calculate the target X, Y, and Z coordinates, as well as the R and A angles.

#### 2.2.3 Surgical procedure

**Figure 1:**
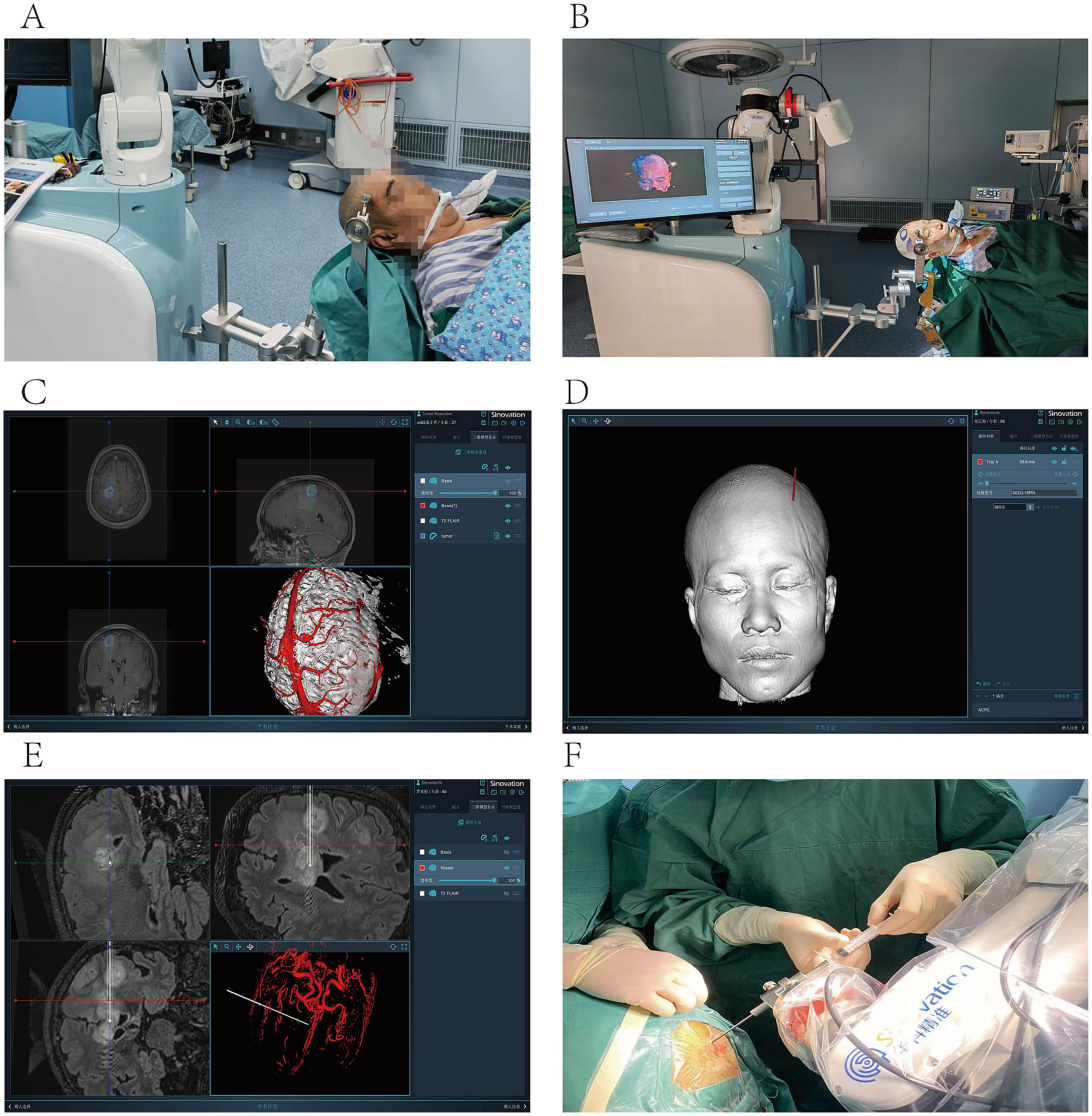
**(A)** After the patient’s head protection part is fixed by the Head immobilization device, push the robot to the front end of the mechanical arm base, which is 50–55 cm away from the midline of the patient’s external auditory canal. **(B)** The locator collects head and facial data from different angles. **(C)** The Huake Precision robot biopsy software integrates preoperative cranial MRI and preoperative localized cranial CT images. **(D)** The reconstruction of head and facial images was successful. **(E)** Calculate the puncture target and the approach to the skull, avoid the sulci and important blood vessels, and take the preoperative target as the objective. **(F)** The robot located the puncture target, successfully inserted a 2.5 mm biopsy needle, and slowly extracted the tissue.

Robot-assisted stereotactic brain biopsy: After successful induction of general anesthesia, the robot was positioned with the distal end of the manipulator base located 50–55 cm from the midline of the patient’s external auditory meatus (Figure 1). Using the base-lift control, the robot was elevated to a stable position until the operating window was aligned with both the patient’s head midline and the manipulator base. The robot was then coupled to the head frame, and the patient’s cranial MRI data were imported into the robotic system (Figure 1). Using the Huake Precision robotic biopsy software f preoperative cranial MRI was fused with the preoperative localization cranial CT images to calculate the target puncture point and intracranial trajectory, while avoiding cortical sulci and major blood vessels. The preoperatively defined target was selected as the operative objective. Preoperative registration was performed by the Huake Precision robot and registration error was computed. Following routine skin preparation and sterile draping, the robot accurately localized the puncture target. A cranial burr hole was created with a surgical drill, and the dura was coagulated with bipolar forceps (Figure 1). A 2.5 mm biopsy needle was smoothly introduced, and tissue samples were obtained sampled by slow withdrawal of the needle while monitoring for active hemorrhage. After achieving hemostasis. Surgical instruments were counted, and the incision was closed (Figure 1).

Frame-based stereotactic brain biopsy (domestic Anke system): Under local anesthesia the Anke frame was installed and CT localization scanning. The obtained data were fused with previously acquired cranial CT images using the surgical planning system to calculate target coordinates (*X*, *Y* and *Z*), ring angle, and arc angle. The patient was then transferred to the operating room and placed in the supine position. After routine skin disinfection, draping, and local anesthesia, a semicircular scalp incision was made. Hemostasis was achieved, a single burr hole was drilled, the dura was coagulated and incised, and the cortical surface was coagulated. A 2.0 mm stereotactic biopsy needle was introduced, and two tissue samples were taken at the target site by rotating the needle 180 degrees to sample two directions. Four additional specimens were taken 0.5 cm above and 0.5 cm below the target. The biopsy needle was left in place for 5 min with no evidence of active bleeding. The scalp incision was closed in layers, a sterile dressing was applied, and the stereotactic frame was removed.

#### 2.2.4 Postoperative management

Excised tissue specimens were subjected to routine pathological examination (11). For all patients, a non-contrast cranial CT scan was performed on postoperative day 1 to calculate the entry-point and target-point errors and to assess for post-biopsy intracranial hemorrhage. Intracranial hemorrhage was defined as follows: a postoperative CT hyperdense area at the biopsy puncture site with a diameter less than 5 mm was not classified as hemorrhage, whereas a hyperdense area greater than 5 mm in diameter on postoperative CT was defined as procedure-related hemorrhage (12).

#### 2.2.5 Data collection

**Figure 2:**
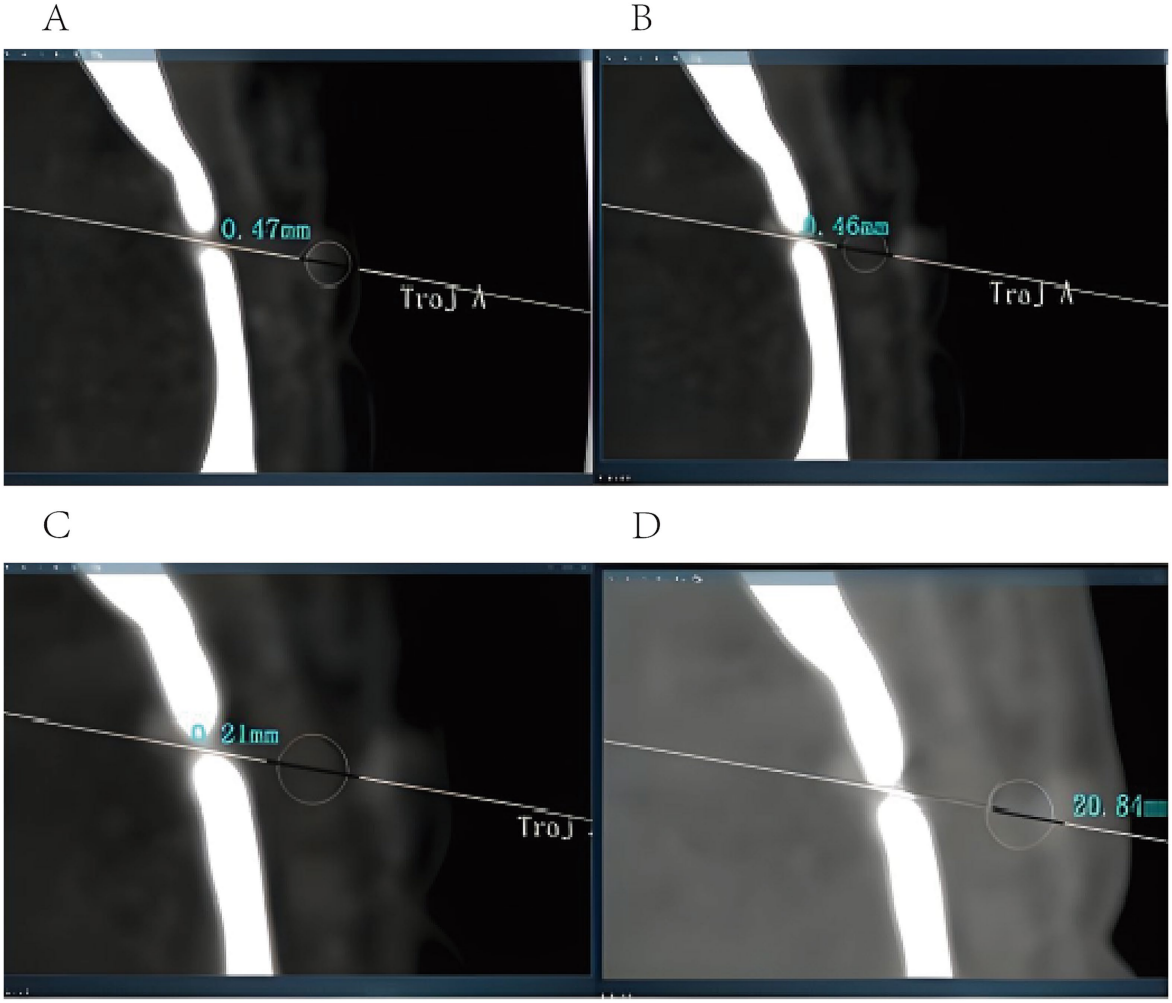
Entry point error calculation: **(A)** the linear deviation between the outermost point of the surgical trajectory and the outermost point of the planned trajectory was 0.47 mm; **(B)** the linear deviation between the midpoint of the surgical trajectory and the midpoint of the planned trajectory was 0.46 mm; **(C)** the linear deviation between the innermost point of the surgical trajectory and the innermost point of the planned trajectory was 0.21 mm. The mean error was 0.38 mm. **(D)** the distance from the midpoint of the surgical trajectory to the target point was 20.81 mm.

**Figure 3:**
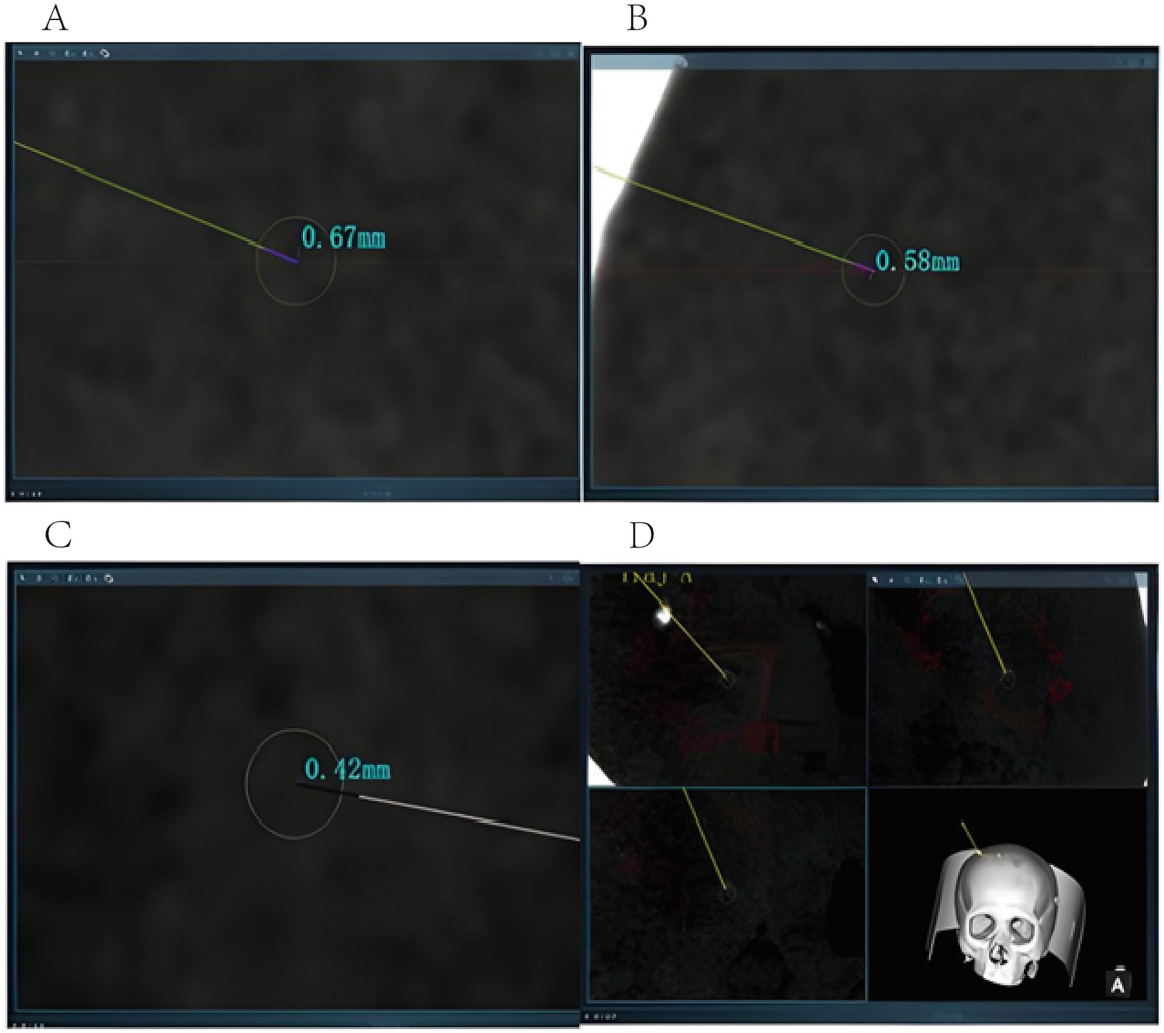
Target error calculation: **(A)** coronal operative target error 0.67 mm; **(B)** sagittal operative target error 0.58 mm; **(C)** axial operative target error 0.42 mm; the mean error was 0.55 mm; **(D)** fusion of the planned surgical trajectory with postoperative CT.

The following variables were collected:3D structured-light facial scan registration error, operative duration, pathology-positive cases, incidence of adverse surgical events, length of hospitalization, surgical costs, entry point error (EPE), and target point error (TPE). EPE was defined as the distance between the planned entry point and the actual entry point. It was determined by fusing postoperative CT data with the preoperatively planned trajectory and measuring the distances between the actual and planned trajectories at the outer table, diploic (central) layer, and the inner table of the skull. The mean of these three measurements was calculated as the EPE (Figure 2). TPE was the distance between the actual surgical target location and the center of the planned target. It was expressed as the mean distance between the biopsy center on postoperative CT images and the planned target across coronal, sagittal, and axial planes, obtained by fusing postoperative CT with preoperative data (Figure 3). The starting point of surgery was defined as the moment when the surgeon began surgical-related operations after successful anesthesia with the patient in the operating room (recorded as T0). This definition criterion excluded time related to anesthesia preparation and preoperative preparation that was not directly relevant to surgical operations, thereby ensuring consistency of operative time measurement between the two groups. The surgical endpoint was defined as the moment when the surgical incision was sutured, the dressing was applied and secured, and the surgeon declared the completion of the surgery (recorded as T1). Operative duration was calculated as T1–T0.

#### 2.2.6 Statistical analysis

All statistical analyses were performed using SPSS 26.0. Continuous variables were expressed as mean ± standard deviation. Categorical variables, including pathology-positive rate and incidence of surgical adverse events, were compared using the chi-square test. Continuous variables—operative duration, surgical cost, and TPE—were analyzed using the nonparametric Wilcoxon test, while EPE was analyzed using the independent-samples *t*-test. A two-sided *p* value < 0.05 was considered statistically significant.

## 3 Results

### 3.1 General data

In the frame-based group, 27 patients (16 males) underwent stereotactic brain biopsy, with a mean age of 53.11 ± 16.43 years (range, 20.0–75.0 years). Among them, 17 cases involved solitary lesions and 25 lesions were supratentorial. In the robot-assisted group, 27 patients (17 males) with a mean age 58.30 ± 12.07 years (range, 20.0–75.0 years) underwent robot-assisted stereotactic brain biopsy. Fourteen cases involved solitary lesions, and 25 lesions were supratentorial. The lesion volume in the robot-assisted group was 9.41 cm^3^ (range, 2.27–20.8 cm^3^), whereas that in the frame-based group was 7.33 cm^3^ (range, 1.38–15.5 cm^3^). The lesion depth in the robot-assisted group was 67.15 ± 6.07 mm, while that in the frame-based group was 54.22 ± 4.16 mm. In terms of past medical history, 6 patients in the robot-assisted group and 4 patients in the frame-based group had a history of hypertension. The blood pressure of all patients was well controlled before surgery, and none had a history of cerebral hemorrhage or coagulation dysfunction. Table 1 presents the specific distribution of biopsy sites and baseline characteristics of the patients.

**Table 1:** Summary of the patient’s clinical characteristics.

Clinical variable	Robot assistance group (*n* = 27)	Frame-based group (*n* = 27)	*p*
Age	58.30 ± 12.07 years (range 20.0–75.0)	53.11 ± 16.43 (range 20.0–75.0)	0.19
Gender			
Male	17	16	0.78
Female	10	11	
Lesion volume	9.41 cm^3^ (range, 2.27–20.8 cm^3^)	7.33 cm^3^ (range, 1.38–15.5 cm^3^)	0.12
Trajectory length	67.15 ± 6.07 mm	54.22 ± 4.16 mm	0.01
Hypertension	6	4	0.57
Biopsy region			
Lobe of the brain	16	19	0.58
Basal ganglia	3	1	
Thalamic	4	2	
Brainstem	1	0	
Corpus callosum	1	2	
Cerebellum	1	2	
Ventricular	1	1	

### 3.2 Registration error of 3D structured-light facial scanning using Huazhong university precision SR1-3D robot Sinovation software

The registration error ranged from 0.22 to 0.51 mm, with a maximum 0.51 mm, minimum 0.22 mm, mean ± standard deviation: 0.31 ± 0.07 mm.

### 3.3 Comparison of operative duration between the two surgical procedures

There was a statistically significant difference in operative time between the robot-assisted surgery group and the frame-based group (*p* = 0.02 < 0.05), with robot-assisted procedures having shorter operative durations than frame-based procedures (Table 2).

**Table 2:** Operative duration of robot-assisted group versus frame-based group.

Group	Duration of the operation
Robot assistance group (*n* = 27)	49.00 ± 15.28
Frame-based group (*n* = 27)	66.07 ± 17.05
*p*	0.02

### 3.4 Comparison of intraoperative pathology positive rates

The histological diagnostic results are presented in Table 3. In the robot-assisted group: pathological diagnoses included gliomas (*n* = 16), embryonal tumors (*n* = 1), meningeal (cranial/spinal) tumors (*n* = 1), lymphoid and hematopoietic system tumors (*n* = 1), metastatic tumors to the central nervous system (*n* = 1), and other (*n* = 6). No cases of choroid plexus tumors, pineal region tumors, cranial and paraspinal nerve tumors, mesenchymal non-meningothelial tumors, germ cell tumors, or sellar region tumors were identified in this group.

**Table 3:** Positive rate of pathological findings in robot-assisted group versus frame-based group.

Group	Clear diagnosis	The diagnosis is unclear.	Total	Positive rate (%)
Robot-assisted group (*n* = 27)	26	1	27	96.29
Frame-based group (*n* = 27)	26	1	27	96.29
Total	52	2	54	96.29

In the frame-based group, pathological diagnoses included gliomas (*n* = 17), lymphoid and hematopoietic system tumors (*n* = 6), and others (*n* = 3). No cases of choroid plexus tumors, embryonal tumors, pineal region tumors, cranial and paraspinal nerve tumors, meningiomas of the brain/spinal cord, mesenchymal non-meningothelial tumors, melanocytic tumors, germ cell tumors, sellar region tumors, or metastatic tumors to the central nervous system were obesrved.

### 3.5 Comparison of the incidence of surgery-related adverse events

In the robot-assisted group, there were three cases of target hemorrhage, accounting for 11.11% of the patients, while two cases occurred in the frame-based group, accounting for 7.40%; all affected patients presented with unilateral limb hemiplegia (Table 4).

**Table 4:** Incidence of postoperative adverse events in robot-assisted group versus frame-based group.

Group	Bleeding in the puncture channel	Target bleeding	Incidence of adverse events (%)
Robot-assisted group (*n* = 27)	0	3	11.11
Frame-based group (*n* = 27)	0	2	7.40
Total	0	5	9.25

### 3.6 Comparison of the length of hospital stay

There was no statistically significant difference in length of hospital stay between the robot-assisted group and the frame-based group (Table 5).

**Table 5:** Length of hospital stay for robot-assisted group versus frame-based group.

Group	Length of hospital stay (day)
Robot-assisted group (*n* = 27)	15.96 ± 9.29
Frame-based group (*n* = 27)	13.62 ± 5.99
*p*	0.412

### 3.7 Comparison of surgical costs

There was a statistically significant difference in operative costs between the robot-assisted group and the frame-based group, with higher surgical expenses in the robot-assisted group (*p* < 0.05) (Table 6).

**Table 6:** Surgical costs of robot-assisted group versus frame-based group.

Group	Surgical costs (CNY)
Robot-assisted group (*n* = 27)	4,009.04 ± 1,278.57
Frame-based group (*n* = 27)	3,304.07 ± 1,047.18
*p*	0.03

### 3.8 Comparison of EPE and TPE

Both EPE and TPE were smaller in the robot-assisted group than in the frame-based group (Table 7).

**Table 7:** Entry point and target point errors for robot-assisted group versus frame-based group.

Group	Entry point error	Target point error
Robot-assisted group (*n* = 27)	0.82 ± 0.37	0.92 ± 0.31
Frame-based group (*n* = 27)	1.06 ± 0.46	1.30 ± 0.44
*p*	0.043	0.001

### 3.9 Illustrated cases

#### 3.9.1 Case 1

**Figure 4:**
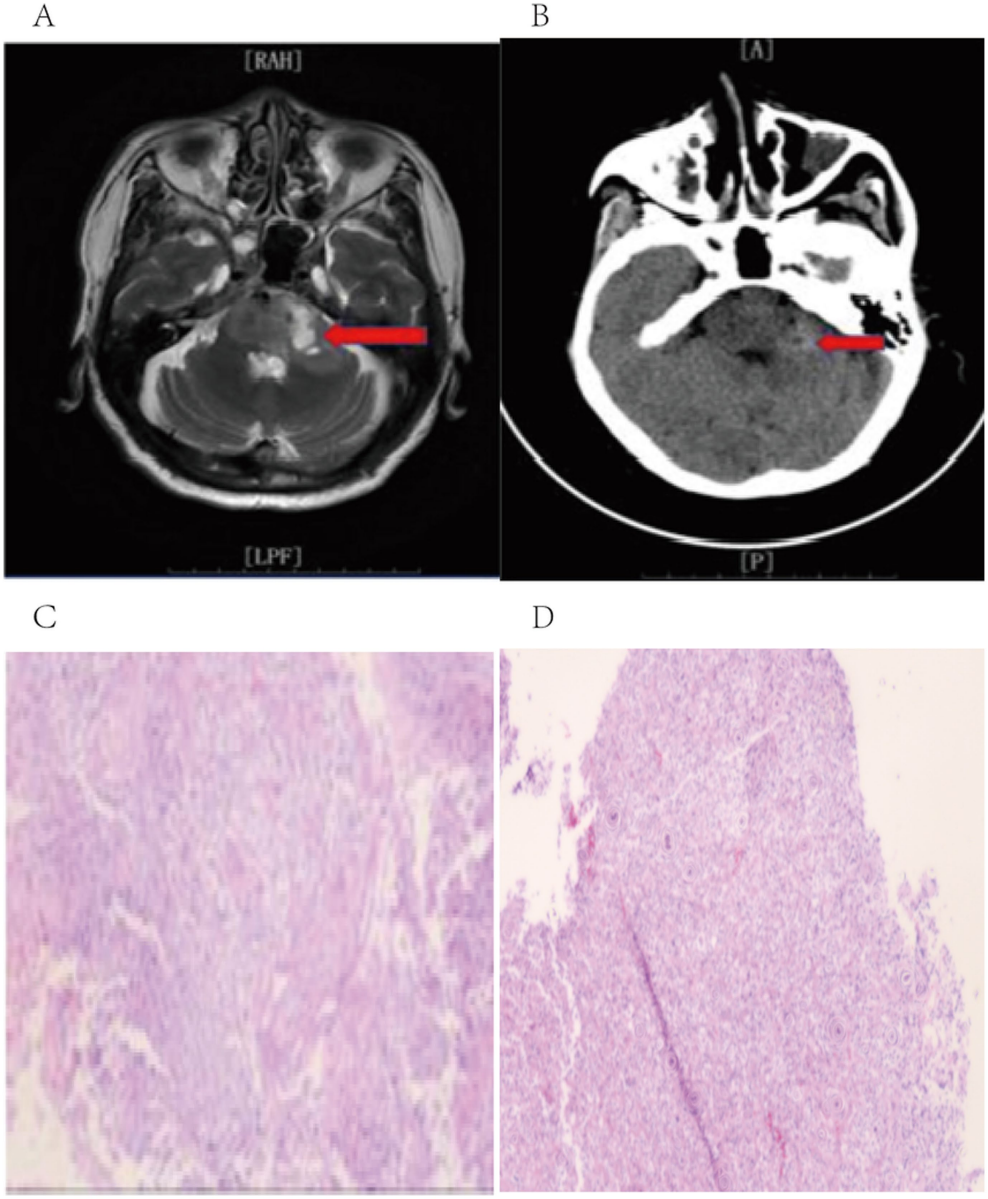
**(A)** Preoperative MRI imaging data; **(B)** Postoperative follow-up CT imaging data; **(C, D)** Postoperative histopathology.

Male, 48 years old, admitted for left facial numbness and generalized weakness of 10 days’ duration. On examination the patient was alert and no obvious abnormality was detected on physical examination. Past medical history was unremarkable. Preoperative cranial MRI demonstrated a mass in the left pontocerebellar angle region; contrast-enhanced sequences showed marked heterogeneous ring enhancement, and FLAIR sequences showed hyperintensity. A malignant brain tumor was suspected on admission, and the patient underwent robot-assisted stereotactic brain biopsy. Diagnostic tissue was successfully obtained, and postoperative histopathology revealed glioblastoma (Figure 4).

#### 3.9.2 Case 2

**Figure 5:**
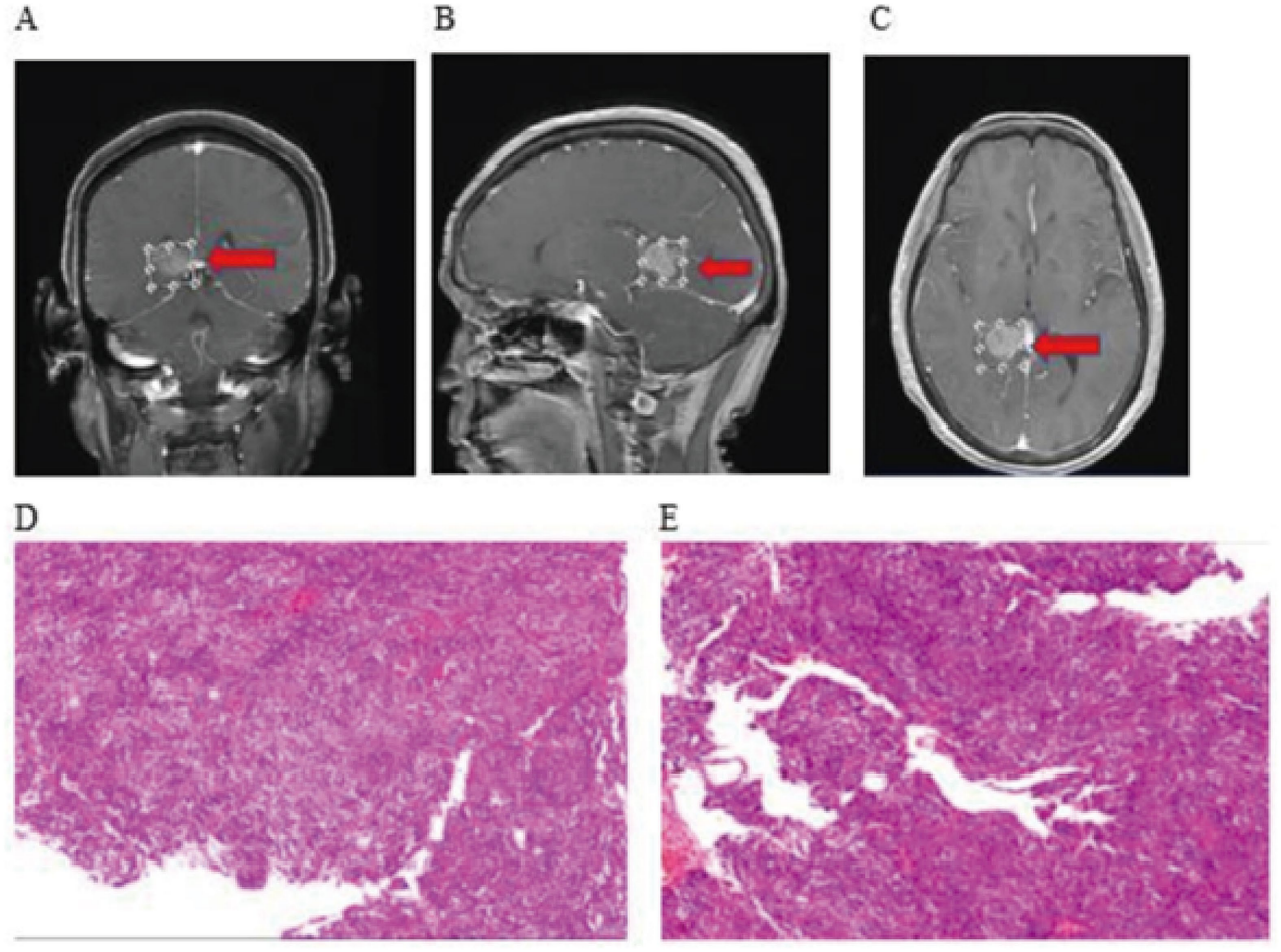
**(A, B, C)** Preoperative MRI images; **(D, E)** Postoperative histopathology.

Female, 58 years old, admitted for dizziness of 7 days’ duration. Neurological examination: patient was conscious and no remarkable abnormalities were detected on physical examination. Past medical history was unremarkable. Cranial MRI on admission demonstrated a lesion adjacent to the posterior horn of the right lateral ventricle; DWI showed slightly increased signal intensity, and contrast-enhanced imaging revealed heterogeneous but marked enhancement. A diagnosis of lymphoma was initially considered; the patient underwent robot-assisted stereotactic brain biopsy, and adequate tissue was obtained. Postoperative histopathology confirmed a psammomatous (or spindle cell) meningioma (Figure 5).

#### 3.9.3 Case 3

**Figure 6:**
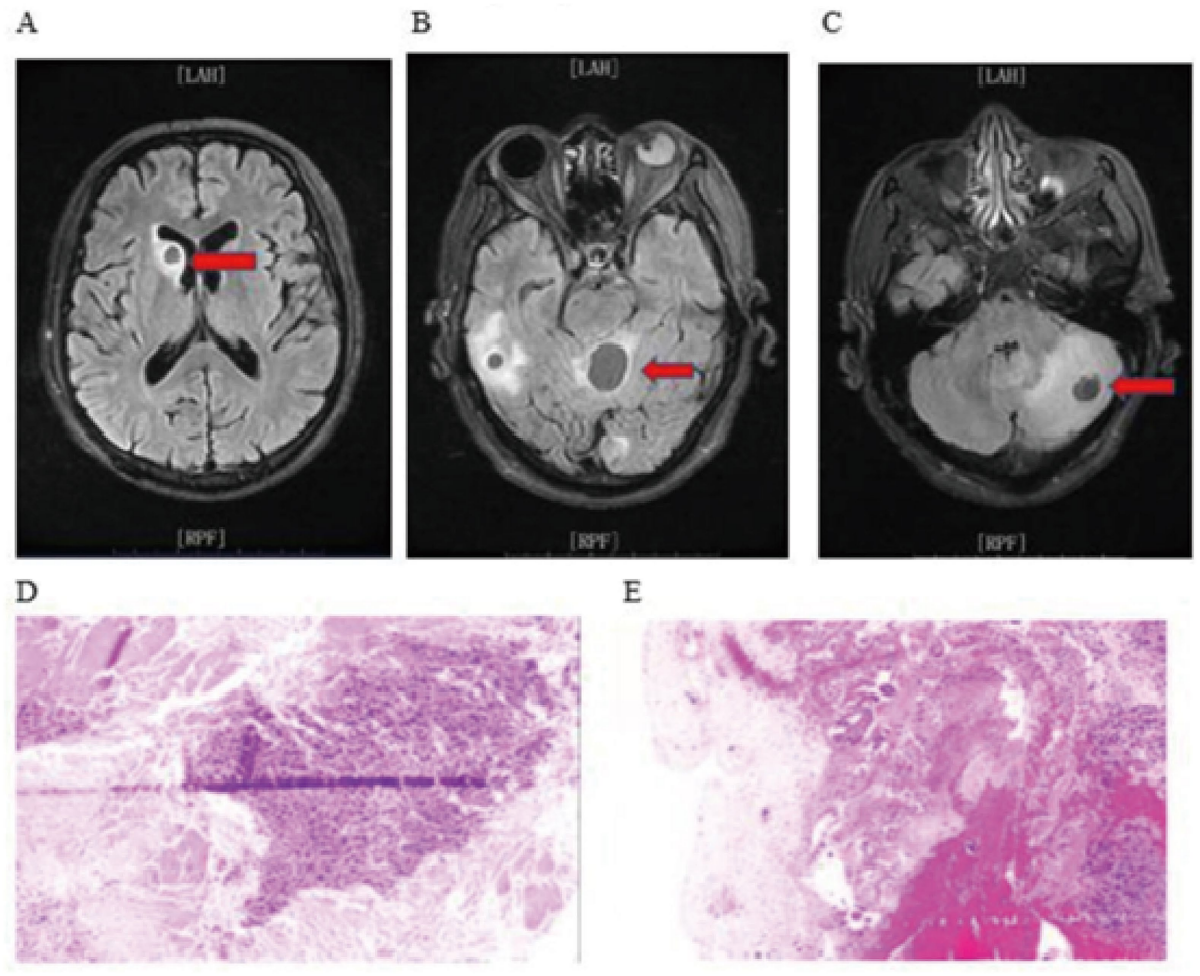
**(A, B, C)** are preoperative MRI images; **(D, E)** are postoperative histopathology.

Male, 63 years old, was admitted to the hospital due to dizziness, headache accompanied by vomiting and unsteady walking for 10 days. Physical examination: Conscious, no obvious abnormalities, and no special past medical history. Upon admission, the head MRI examination indicated multiple space-occupying lesions in the brain, suggesting metastatic tumors. Multiple nodular long T1 and T2 signal shadows could be seen in the right ventricle, left parietal lobe, right temporal lobe, left occipital lobe, left cerebellar hemisphere, and superior vermis of the cerebellum. The signals were uniform, with clear boundaries. The largest one was located in the superior vermis of the cerebellum, measuring 26 × 20 mm. Small patchy edema signal shadows could be seen around. Upon admission, metastatic tumors were considered. Robot-assisted stereotactic brain biopsy was performed, and pathological tissues were successfully obtained. The postoperative pathology was poorly differentiated metastatic carcinoma of the cerebellum (Figure 6).

#### 3.9.4 Case 4

**Figure 7:**
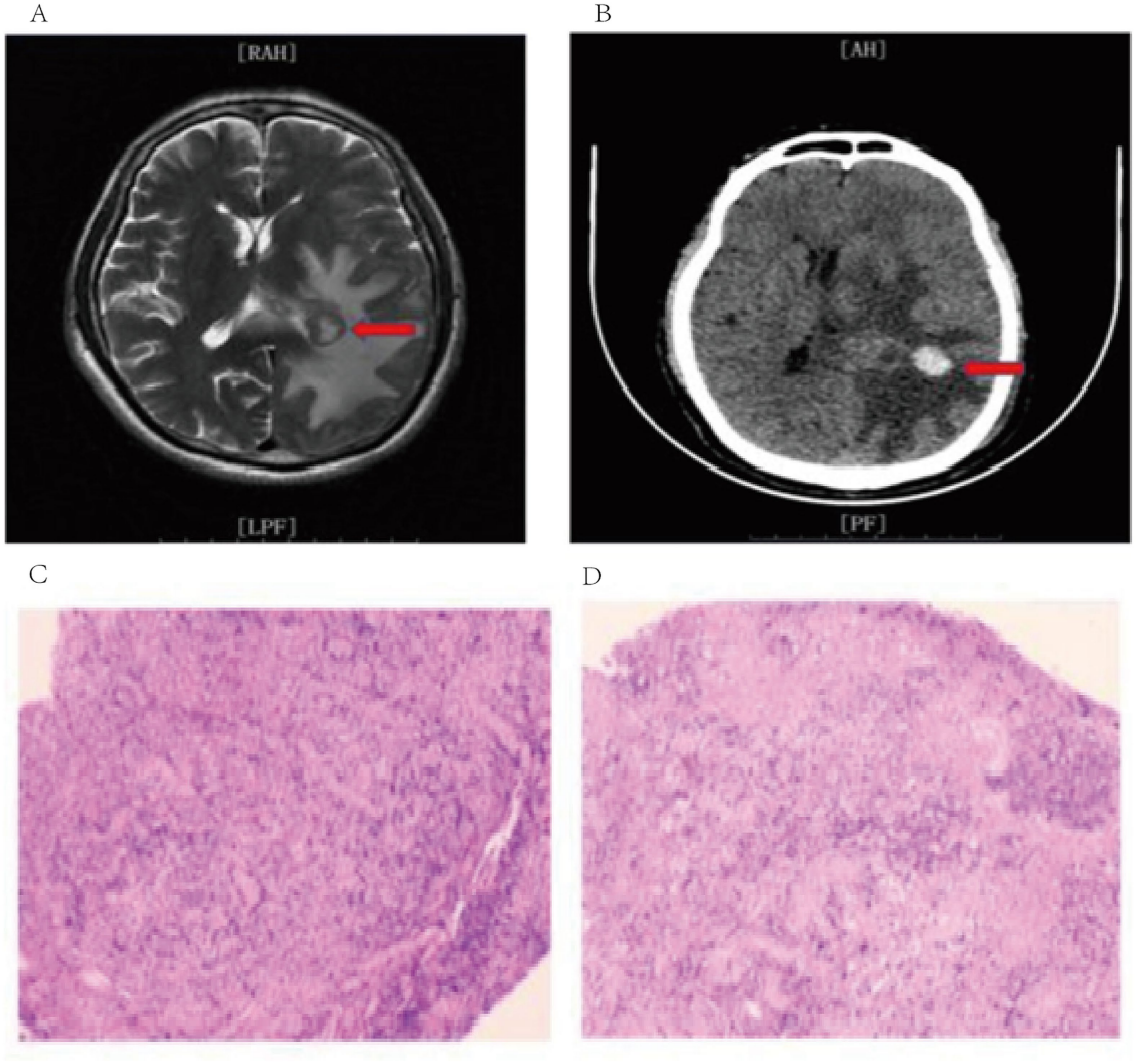
**(A)** Preoperative MRI imaging data; **(B)** Postoperative CT imaging data. **(C, D)** Postoperative pathology.

Male, 67 years old, was admitted to the hospital due to slurred speech accompanied by unsteady walking for over half a month, which worsened 1 day. Physical examination revealed clear consciousness, incoherent responses, slurred speech, weakened calculation, memory, comprehension and orientation abilities. No other abnormalities were found in other physical examinations, and there were no special past medical history. Upon admission, a complete MRI examination indicated that there was a space-occupying lesion in the posterior horn of the left lateral ventricle with surrounding subfoci forming. High-grade glioma was considered possible, and lymphoma was not excluded. A nodular shadow approximately 20 × 25 mm in size could be seen beside the posterior horn of the left lateral ventricle. Enhanced scanning showed a ring-shaped enhancement, and large patchy edema bands could be seen around it. Robot-assisted stereotactic brain biopsy was performed, and pathological tissues were successfully obtained. The postoperative pathology was “occipital lobe” non-specific diffuse large B-cell lymphoma (Figure 7).

## 4 Discussion

Intracranial tissue biopsy is a safe and effective method for characterizing intracranial space-occupying lesions, as the specimen obtained intraoperatively provide pathological evidence for diagnosis and treatment (13–15). With advances in neurosurgery and oncology, stereotactic brain biopsy has become an important tool for establishing the diagnosis of intracranial lesions (16).

In this study, a retrospective analysis of 54 cases undergoing stereotactic brain biopsy was conducted. It was found that the robot-assisted group showed more advantages over frame-based group in terms of operation duration and accuracy. There were no significant differences in biopsy success rate or incidence of adverse events between the two groups. However, the cost of robot-assisted group was higher, mainly due to equipment-related factors and anesthesia fees.

### 4.1 Accuracy of robot-assisted stereotactic brain biopsy

Improving the positive diagnosis rate of stereotactic brain biopsy is a key factor influencing the surgical outcomes. The positive diagnosis rate is affected by multiple factors. Yan Li et al. concluded a multivariate analysis of 584 patients that the smaller and concluded that smaller TPE, larger lesion volume, and higher enhancement ratio of intracranial lesions in MRI images were associated with higher positive diagnosis rates (17). Other studies have similarly also suggested that TPE I plays an important role in improving biopsy positivity (18, 19). Among these factors, TPE is the only one that can be improved through surgical methods.

Numerous studies have confirmed the reliability of robot-assisted biopsy in target localization. In 700 biopsy surgeries using the Remebot system, the EPE was 0.99 ± 0.24 mm (range, 0.56–1.73 mm), and the TPE was 1.13 ± 0.30 mm (range, 0.57–1.78 mm) (13). Using the RONNA G4 system, the TPE in surgery was 1.95 ± 1.11 mm, and the EPE was 1.42 ± 0.74 mm (20). In electrode implantation surgeries using ROSA, the EPE was 1.59 ± 1.1 mm, and the TPE was 2.22 ± 1.71 mm [8]. When with the iSYS 1 robot, the median EPE was 1.3 mm, and the median TPE was 0.9 mm (21), while the Neuromate robot showed a median TPE of 2.7 mm (range, 0.5–4.2 mm) (22). Other types of surgical robots, including Surgiscope (23), MKM (24), and Neuromate (16), have all demonstrated reliable precision in robot-assisted surgeries.

However, few studies have directly compared the EPE and TPE between 3D structured light robot-assisted stereotactic brain biopsy and frame-based stereotactic surgery. This study compared the entry point errors and target point errors before and after surgery and found that the entry point error of the robot group was significantly lower than that of the frame group (0.82 ± 0.37 vs. 1.06 ± 0.46, *p* < 0.05). Similarly, the target point error of the robot-assisted group was significantly lower than that of the frame-based group (0.92 ± 0.31 vs. 1.30 ± 0.44, *p* < 0.05).

It should be acknowledged that a difference of 0.3–0.4 mm may not represent a decisive clinical advantage. In routine cases with large, superficially located, and well - defined lesions, the clinical benefits of this improvement may be limited. Moreover, due to the relatively limited sample size, the observed associations could not be further validated. Although there were no significant difference in positive rates between the two groups, evidence from previous studies suggests that improved, target point accuracy is likely beneficial for improving biopsy positivity. Confirmation of this hypothesis requires multi-center studies with larger sample sizes.

Before frame-based brain stereotactic biopsy, a complex process of head-frame installation and trajectory planning is required. Positioning parameters are complex and require manual adjustment. Repeated use of positioning instruments may result in wear, and displacement of the head-frame during patient transfer, especially in cases with multiple lesions, necessitating repeated parameter recalibration for each target. All these factors may lead to a decrease in accuracy. In contrast, robot-assisted procedures achieve a higher degree of automation. High-precision robotic arms and integrated surgical channels simplify operative steps and minimize errors related to manual operation and instrument wear. Meanwhile, the automation of robot-assisted surgery enables repeated operations along the same trajectory with consistent accuracy, avoiding errors caused by hand tremors (9).

### 4.2 Positive rate of robot-assisted stereotactic brain biopsy

The biopsy success rate in the robot-assisted group in this study was 96.29%, which was comparable to that of the frame-based group, with no statistically significant difference between the two. A previous study has reported diagnostic yields for robot-assisted stereotactic biopsy ranging from 75 to 100% (20). The diagnostic yields for CRW stereotactic frame-based and frameless stereotactic biopsies have been reported as 95.2 and 89.4%, respectively (25); the diagnostic yield of ISG surgical robot brain biopsy is 89% (26). In a comparison between Remebot robot-assisted and frame-based stereotactic brain biopsies, the diagnostic yields were 91.4% and, respectively 93.5% (27).

Multiple factors influence the positive rate of pathological biopsy. For example, intraoperative squash smear has been shown to reduce the risk of false-negative biopsy results from 11.1 to 3.7% (28). Compared with frozen section, intraoperative squash smear offers several advantages: it is faster, requires less tissue, thereby preserving more material for histopathological examination and molecular analysis; it allows evaluation of thin glial processes or neuronal cytoplasmic extensions and facilitates identification of tumor cell glial or neuronal phenotypes, reactive astrocytes, and a fibrous background; and it renders nuclear details more conspicuous. The choice of biopsy needle may also affect histological yield. The literature has reported that specimens obtained with higher vacuum pressure or with the Laitinen needle are more suitable for histopathological assessment and contribute to improved diagnostic success rates (29). For patients with an initial negative biopsy, a second biopsy is often required. In addition, preoperative use of corticosteroids may lead to nondiagnostic results; therefore, such medications should be avoided prior to biopsy whenever possible (30).

### 4.3 Efficiency of robot-assisted stereotactic brain biopsy

Compared with conventional frame-based stereotactic biopsy, robot-assisted biopsy requires a shorter operative time. In this study, the mean operative duration in the robot-assisted group was significantly shorter than that in the frame-based group (49.00 ± 15.28 vs. 66.07 ± 17.05 min). This difference can be attributed to the workflow of robot-assisted stereotactic biopsy, which involves importing the planned trajectory data into the operating platform and performing a simple 3D structured-light scan prior to the procedure. This workflow is particularly advantageous for deep-seated lesions such as those located in the cerebellum or brainstem, as it is less affected by patient positioning. By contrast, frame-based biopsy requires verification of parameters for each target and manual coordinate calculations, and is more susceptible to procedural delays due to human factors, all of which contribute to longer operative time. These issues are especially relevant in cases of multifocal intracranial lesions, which necessitate frame installation and removal as well as changes in patient positioning. Moreover, frame-based stereotactic brain biopsy requires close intraoperative coordination among surgeons; when preoperative preparation time is considered, frame-based procedures consume even more time. On the day of surgery, the head frame must be fixed to the patient’s skull for CT scanning, after which the patient is transferred back to the operating room to await determination of target parameter determination. In contrast, robot-assisted stereotactic biopsy is more streamlined: the surgeon designs stereotactic trajectories using the robot planning software based on preoperative MRI and imports the preoperative plan into the surgical robot intraoperatively, thereby shortening the overall operating time. Published reports are consistent with these findings: ROSA frameless robotic biopsies have been reported with operative times of 25 ± 15 min (31), and RONNA G4 robotic brain biopsies with a mean operative time of 64.62 ± 19.05 min (20), aligning with the robot-assisted stereotactic biopsy durations observed in this study.

### 4.4 Safety of robot-assisted stereotactic brain biopsy

The most common complications of stereotactic biopsy include target-site hemorrhage and trajectory hemorrhage, followed by failure to obtain diagnostic tissue; other complications include infection, neurological deficits, and seizures (27). When clinical signs have already manifested, the probability of intracranial hemorrhage can be as high as 60% (32). If no diagnostic tissue is obtained, a second biopsy is required (30). In this study intracranial hemorrhage was considered a postoperative adverse event. The incidence of adverse events was 11.11% in the robot-assisted group and 7.40% in the frame-based group, indicating comparable adverse event rates between the two approaches. In all cases with intracranial hemorrhage, the bleeding was confined to the lesion (target-site hemorrhage) and no hemorrhage along the biopsy trajectory was observed. Clinically, patients presented with unilateral motor paresis. These findings partly support that preoperative imaging reduces the risk of injuring cortical sulcal vessels or bridging veins. These findings partly support that careful preoperative imaging may reduce the risk of injury to cortical sulcal vessels or bridging veins. Robot-assisted surgery appears to demonstrate a favorable safety profile; however, due to the limited sample size, it cannot be definitively stated that its safety surpasses that of traditional frame-based techniques.

The following circumstances may increase the likelihood of procedural hemorrhage during stereotactic biopsy. According to the literature, the risk of bleeding after stereotactic biopsy of malignant gliomas can be as high as 59% (12); platelet counts below 150 × 10^9^/L are associated with a biopsy-related bleeding rate of 8% (33); lesions located in the brainstem carry a bleeding risk of approximately 7% during biopsy (34); diabetes, thalamic lesions, and basal ganglia lesions have been associated with a biopsy bleeding rate of 9% (35); a prothrombin time longer than 12.7 s in patients with malignant glioma is associated with a biopsy bleeding rate of 31.3% (36). Lesions with a diameter of 3 cm or less have been reported to have a biopsy bleeding rate of 19% (37). In addition, manipulation of the biopsy needle during tissue retrieval and trajectory adjustments during when planning may increase the risk of tract hemorrhage after the procedure (9), and postoperative intracranial bleeding may also be related to human error in trajectory planning (38).

To reduce the risk of postoperative intracranial hemorrhage, the use of an improved biopsy needle may be beneficial. Such needle features a smooth, rounded tip that, can displace blood vessels laterally upon contact, thereby reducing the likelihood of vascular injury.

### 4.5 Comfort of robot-assisted stereotactic brain biopsy

This study compared operative durations between robot-assisted and conventional frame-based stereotactic biopsies, demonstrating the efficiency of robotic systems in stereotactic procedures. Beyond operative time, frame-based systems such as the Anke head frame impose additional burdens: Although local anesthesia is used during frame placement, the heavy metal apparatus may still cause unnecessary discomfort for patients and their families (16). By contrast, the Huake Precision SR1-3D surgical robot requires only the importation of MRI images into the surgical planning system, trajectory definition, and transfer of the plan to the robot. After a 5–10 min 3D structured-light scan for registration, the procedure can be initiated. This streamlined workflow, reduces patient discomfort, and provides a more comfortable patient experience.

### 4.6 Flexibility of robot-assisted stereotactic brain biopsy

In this study, complex cases were encountered, including lesions such as deep-seated intracranial lesions and posterior cranial fossa lesions, and multiple lesions requiring procedures at two or more sites. Frame-based stereotactic biopsy necessitates patient repositioning and repeated disassembly and reattachment of the head frame, whereas surgical robots eliminate these inconveniences. In addition, skull size—small in pediatric patients and large in some adults—may affect frame mounting. It has been reported that surgical robots offer greater flexibility than conventional frame-based stereotactic brain biopsy for both pediatric and adult patients with varying adult skulls (39). Moreover, when addressing mesial temporal or posterior fossa lesions, procedures performed with conventional frames are technically more demanding, a challenge that is less pronounced with robotic systems (21).

### 4.7 Surgical costs of robot-assisted stereotactic brain biopsy

The total cost of brain biopsy encompasses all medical expenses directly associated with the surgical procedure during the patient’s hospital stay. This primarily includes the following key cost components. The cost of a brain biopsy procedure is approximately 1,600 yuan, and this expense is comparable between the two patient groups. Allocation of capital costs and anesthesia expenses differs between groups. The robot-assisted group incurs additional costs due to the purchase and maintenance of the robotic system, which adds approximately 500 yuan to the cost per patient. In contrast, the frame-based group does not face such capital expenses. Regarding anesthesia: The robot-assisted group requires brief general anesthesia, costing approximately 400 yuan, whereas the frame group only requires local anesthesia, thus avoiding this expense. Both groups incur similar costs for imaging examinations, hospitalization, medications, puncture needles, sterilization frame covers, and disposable cranial drill bits, totaling approximately 1,500 yuan. In summary, the primary cost differences for the robot-assisted group mainly stem from “capital cost allocation and anesthesia expenses.”

### 4.8 Limitations

This study is a single-center retrospective investigation with a small sample size, and therefore, cannot avoid methodological limitations and insufficient statistical power. Consequently, future studies should involve prospective cohort studies conducted collaboratively across multiple centers. Additionally, increasing the sample size is essential to enhance the statistical power of the analysis. Operative duration may be influenced by the surgeon’s experience; this study did not account for the operators’ learning curves for the two surgical techniques. In future studies, we plan to investigate and compare the learning curves of both approaches.

## 5 Conclusion

Robot-assisted surgery offers higher precision and is probably advantageous for diagnosing deep-seated and small intracranial lesions.3D structured-light robotic systems robots offer greater flexibility in different application scenarios. There were no significant differences between robot-assisted surgery and frame-based surgery in terms of length of hospital stay, postoperative adverse events, or hospitalization costs, although robot-assisted procedures incurred higher surgical expenses. 3D structured-light robotic systems can shorten operative time and improve surgical efficiency.

## Data Availability

The original contributions presented in the study are included in the article/supplementary material, further inquiries can be directed to the corresponding authors.
